# Reliable heritability estimation using sparse regularization in ultrahigh dimensional genome-wide association studies

**DOI:** 10.1186/s12859-019-2792-7

**Published:** 2019-04-30

**Authors:** Xin Li, Dongya Wu, Yue Cui, Bing Liu, Henrik Walter, Gunter Schumann, Chong Li, Tianzi Jiang

**Affiliations:** 1 School of Mathematical Sciences, Zhejiang University, 38 Zheda Road, Hangzhou, 310027 China; 2 Brainnetome Center, Institute of Automation, Chinese Academy of Sciences, 95 East Zhongguancun Road, Beijing, 100190 China; 3 National Laboratory of Pattern Recognition, Institute of Automation, Chinese Academy of Sciences, 95 East Zhongguancun Road, Beijing, 100190 China; 4 CAS Center for Excellence in Brain Science and Intelligence Technology, Institute of Automation, Chinese Academy of Sciences, 95 East Zhongguancun Road, Beijing, 100190 China; 5 The Clinical Hospital of Chengdu Brain Science Institute, MOE Key Lab for Neuroinformation, University of Electronic Science and Technology of China, 4 Section 2 North Jianshe Road, Chengdu, 610054 China; 6 The Queensland Brain Institute, University of Queensland, Brisbane, QLD 4072 Australia; 7 University of Chinese Academy of Sciences, 19 Yuquan Road, Beijing, 100049 China; 8 Department of Psychiatry and Psychotherapy, Campus Charité Mitte, Charité, Universitätsmedizin Berlin, Berlin, Germany; 9 Centre for Population Neuroscience and Stratified Medicine (PONS) and MRC-SGDP Centre, Institute of Psychiatry, Psychology & Neuroscience, King’s College London, London, United Kingdom

**Keywords:** Heritability, Reliable estimation, Sparse regularization, Standard error, Simulation

## Abstract

**Background:**

Data from genome-wide association studies (GWASs) have been used to estimate the heritability of human complex traits in recent years. Existing methods are based on the linear mixed model, with the assumption that the genetic effects are random variables, which is opposite to the fixed effect assumption embedded in the framework of quantitative genetics theory. Moreover, heritability estimators provided by existing methods may have large standard errors, which calls for the development of reliable and accurate methods to estimate heritability.

**Results:**

In this paper, we first investigate the influences of the fixed and random effect assumption on heritability estimation, and prove that these two assumptions are equivalent under mild conditions in the theoretical aspect. Second, we propose a two-stage strategy by first performing sparse regularization via cross-validated elastic net, and then applying variance estimation methods to construct reliable heritability estimations. Results on both simulated data and real data show that our strategy achieves a considerable reduction in the standard error while reserving the accuracy.

**Conclusions:**

The proposed strategy allows for a reliable and accurate heritability estimation using GWAS data. It shows the promising future that reliable estimations can still be obtained with even a relatively restricted sample size, and should be especially useful for large-scale heritability analyses in the genomics era.

**Electronic supplementary material:**

The online version of this article (10.1186/s12859-019-2792-7) contains supplementary material, which is available to authorized users.

## Background

Heritability measures how much the variation of a phenotypic trait in a population is caused by the genetic variation among individuals in that population. It has two specific types of definition: the broad sense and the narrow sense. The narrow-sense heritability is of more importance in genetic applications, which is defined as the ratio of the additive genetic variance to the total phenotypic variance [1]. With the tremendous technological advances in genome-wide association studies (GWASs) in the last few decades, hundreds of thousands of genetic markers for individuals have been discovered, usually single nucleotide polymorphisms (SNPs), aiming to explore the genetic architecture of human complex traits. Heritability based on GWASs, termed as the SNP heritability [2], has been serving as a more and more critical measure in this exploration, and can guide downstream analysis on more specific biological questions. Hereinafter, we consider the SNP heritability unless otherwise specified.

Traditional approaches to estimating narrow-sense heritability are based on twin or pedigree studies, in which genetic variance can be estimated from phenotypic similarity between relatives; see, e.g., [1, 3] and references therein. But in practice, it is rather difficult to completely partition the genetic variance from the variance resulted from shared common environmental factors, as relatives often share similar genes and are more likely to be raised in similar environment [4]. In modern GWASs, designs based on a population sample of unrelated people help to overcome the confounding of genes and environment, with the SNP heritability being viewed as a lower bound for the narrow-sense heritability. However, for most traits the declared highly significant SNPs fail to capture all the genetic variance; see, e.g., [5, 6]. This has been referred to as the “missing heritability” problem [7, 8]. To address this gap, researchers in [9] developed the software genome-wide complex trait analysis (GCTA) to estimate the SNP heritability without the requirement that individual SNPs are significant, arriving at a higher lower bound for the narrow-sense heritability [10]. Recently, computing tools such as BOLT-REML [11], BayesR [12], and massively expedited genome-wide heritability analysis (MEGHA) [13] have been developed to achieve a higher speed. These works make use of the linear mixed model (LMM) to consider all SNPs across the genome-wide average, assuming that the genetic effects are random variables and the genotypes are fixed quantities.

However, from the framework of quantitative genetics theory, the effects of genetic markers on a trait are fixed quantities, and genetic variance stems from variation at quantitative trait locus (QTL) genotypes [1, 14]. What is the difference between the fixed and random effect assumption? Does it matter which assumption is used to estimate heritability? This motivates us to investigate the two assumptions in order to compare their influences on heritability estimation. Moreover, heritability estimators produced by GCTA and following tools may have large standard errors, which is especially the case in the field of imaging genetics, where the sample size cannot increase arbitrarily due to high costs; see, e.g., [15–17]. This stimulates the main focus of our work to construct reliable estimators for heritability with smaller standard errors in the ultrahigh dimensional scenario. The main contributions of this paper are as follows. First, we investigate the influences of the fixed and random effect assumption on heritability estimation, and prove that these two assumptions are equivalent under mild conditions in the theoretical aspect. Second, former GWASs have pointed out that the number of SNPs with nonzero effects that are associated with a given disease or a trait may be relatively small or moderate (e.g., ∼10^3^), though the whole number of SNPs is usually very large (e.g., 10^5^∼10^6^) [18, 19]. In other words, not all SNPs are causal (strictly speaking, here “causal SNPs” just refer to SNPs with nonzero effects), or at least not all SNPs are in perfect linkage disequilibrium (LD) with QTL. In a statistical terminology, the underlying true model is sparse. Therefore, we make use of the underlying sparse structure of GWAS data, and propose a two-stage strategy by first performing sparse regularization via cross-validated elastic net and then applying certain variance estimation methods to construct reliable heritability estimations. Results from simulated data and real neuroanatomical data from the IMAGEN project show that our strategy can provide estimators with a considerable reduction in the standard error while retaining the accuracy. The results demonstrate the promising capability of our strategy for large-scale heritability analyses in the genomics era, especially in the field of imaging genetics, where the sample size is usually limited nowadays.

## Methods

We begin this section by first introducing some definitions and notations for future reference. For 0<*q*<+*∞*, the *ℓ*_*q*_ norm of a vector $u\in \mathbb {R}^{n}$ is defined as $\|u\|_{q}:=\left (\sum _{i=1}^{n} |u_{i}|^{q}\right)^{1/q}$. We say that *u*=**0** if *u*_*i*_=0 for all *i*=1,2,⋯,*n*. For *m*≥1, let $\mathbb {I}_{m}$ stand for the *m*×*m* identity matrix. For a matrix $W\in \mathbb {R}^{m\times n}$, we use *W*_*ij*_ (*i*=1,2,⋯,*m*,*j*=1,2,⋯,*n*) to denote its *ij*-th entry, *W*_*i*·_ (*i*=1,2,⋯,*m*) to denote its *i*-th row, and *W*_·*j*_ (*j*=1,2,⋯,*n*) to denote its *j*-th column. For any index set *M*⊆{1,2,⋯,*n*}, we use *u*_*M*_ to denote the subvector containing the components of the vector *u* that are indexed by *M*, and *W*_*M*_ to denote the submatrix containing the columns of the matrix *W* that are indexed by *M*.

### Model

In this paper, we consider the following sparse linear model to approximate the true underlying model in GWASs,

1 $$ y=Wu^{*}+e,  $$

where $y\in \mathbb {R}^{m}$ is a vector of observations, *W* is an *m*×*n* (*m*≪*n*) design matrix storing the SNP information, $u^{*}\in \mathbb {R}^{n}$ is the unknown vector representing the SNP effects with *s* (*s*≤*n*) nonzero entries, and *e* is a vector of residual effects with $e\sim \mathcal {N}(0,\sigma _{e}^{2}\mathbb {I}_{m})$. The true model is denoted as $M_{0}:=\{j:u^{*}_{j}\neq 0\}$. Then the cardinality of the true model |*M*_0_|=*s* represents the number of causal SNPs of a given trait. The sparsity level is defined as *γ*:=*s*/*n*, which may be high or low according to the trait studied. When there are other covariates (such as overall mean, sex and age) to be considered, we simply apply the method proposed in [20], which projects out the nuisance variables (covariates).

Then we state two assumptions regarding the model Eq. 1.

*Fixed Effect Assumption.* This assumption is consistent with the quantitative genetics paradigm. We now specify it in the sparse scenario as follows: (i). The rows of the design matrix *W*_1·_,*W*_2·_,⋯,*W*_*m*·_ are independent and identically distributed random vectors with mean $\mathbb {E}(W_{1 \cdot })=\mathbf {0}$ and *n*×*n* positive definite covariance matrix *Σ*=Cov(*W*_1·_); (ii). The residuals *e*_1_,*e*_2_,⋯,*e*_*m*_ are independent of the design matrix *W*; (iii). The vector *u* consists of fixed quantities with supp(*u*^∗^)=*M*_0_. Here the assumed covariance structure of *W*_*i*·_ is used to characterize the correlations between the *n* SNPs.

*Random Effect Assumption.* Recently, researchers in [9, 10] made use of this assumption to solve the “missing heritability” problem. We also endow it with the sparse structure as follows: (i). $\{u^{*}_{j}:j\in M_{0}\}$ are a set of independent and identically distributed Gaussian random variables with mean 0 and variance $\sigma _{u}^{2}$; (ii). For any *i*∈{1,2,·,*m*} and *j*∈*M*_0_, *e*_*i*_ is independent of $u^{*}_{j}$; (iii). The design matrix *W* is made up with fixed entries.

We now describe *W* in detail under the context of genetics. Noting from the facts that in GWAS each SNP is regarded as a binomial random variable with two trials, and that the success probability is defined as “reference allele frequency”, the entries of the design matrix *W* can be formulated by another matrix *Z* in the following way:

2 $$ W_{ij}=\frac{Z_{ij}-2p_{j}}{\sqrt{2p_{j}(1-p_{j})}},\ i=1,2,\cdots,m,\ j=1,2,\cdots,n,  $$

where the matrix *Z* stores the original genetic information in a population. Concretely speaking, the genotype of each SNP is coded in this way: *Z*_*ij*_=0 (resp. 1, resp. 2) if the genotype of the *i*^*th*^ individual at locus *j* is bb (resp. Bb, resp. BB), and *p*_*j*_ is the frequency of the reference allele at locus *j*. After being constructed as above, *W* is the standard genotype matrix with each column/row standardized to have zero mean and unit variance.

Then we are at the stage to define heritability under the two assumptions on the model Eq. 1. Recall the definition that heritability measures the fraction of variation of a given trait that can be explained by variation of genetic markers among individuals in a population. For the fixed effect assumption, let $\tau ^{2}=u^{*T}\Sigma u^{*}=\left \|\Sigma ^{1/2}u^{*}\right \|_{2}^{2}$, which represents a measure of total genetic variance attributed to causal SNPs. With the residual variance $\sigma _{e}^{2}=\text {Var}(e_{i})$, we can naturally define the heritability as the proportion of explained variance in the linear model Eq. 1:

3 $$ h_{\text{fixed}}^{*}=\frac{\tau^{2}}{\tau^{2}+\sigma_{e}^{2}}.  $$

For the random effect assumption, which has been investigated by many authors [9, 21], the heritability is defined as:

4 $$ h_{\text{rand.}}^{*}=\frac{s\sigma_{u}^{2}}{s\sigma_{u}^{2}+\sigma_{e}^{2}}.  $$

The following proposition tells us that Eq. 3 is equivalent to Eq. 4 under the assumption that the nonzero genetic effects $\{u^{*}_{j}:j\in M_{0}\}$ are independently drawn from a prior distribution. Under this assumption, in order to guarantee that the total genetic variance *τ*^2^ is still a fixed quantity, we make a slight modification to take expectation over the distribution of *u*^∗^, that is, $\tau ^{2}=\mathbb {E}_{u^{*}}\left (u^{*\top }\Sigma u^{*}\right)$.

#### **Proposition 1**

Suppose that the nonzero genetic effects $\{u^{*}_{j}:j\in M_{0}\}$ are independently drawn from a prior distribution with mean 0 and variance ${Var}(u_{j}^{*})=\sigma _{u}^{2}$, and that for any *i*∈{1,2,⋯,*m*}, $W_{i \cdot }^{\top }$ and *u*^∗^ are independent. Then $h_{{fixed}}^{*}=h_{{rand.}}^{*}$.

#### *Proof*

For any *i*∈{1,2,⋯,*m*} fixed, the total genetic variance attributed to causal SNPs is □

5 $$ {\begin{aligned} \tau^{2}&\,=\,\mathbb{E}_{u^{*}}\left(u^{*\top}\Sigma u^{*}\right)\,=\, \mathbb{E}_{u^{*}}\left[u^{*\top}\text{Cov}(W_{i \cdot})u^{*}\right]\,=\,\mathbb{E}_{u^{*}}\left[u^{*\top}\mathbb{E}_{W_{i \cdot}}({W_{i \cdot}}^{\top} W_{i \cdot})u^{*}\right]\\ &=\mathbb{E}\left(W_{i \cdot}u^{*}\right)^{2}=\mathbb{E}_{W_{i \cdot}}\left[\mathbb{E}_{u^{*}}(W_{i \cdot}u^{*})^{2}|W_{i \cdot}\right], \end{aligned}}  $$

where the third equality is from the fixed effect assumption (i) that $\mathbb {E}_{W_{i \cdot }}(W_{i \cdot })=\mathbf {0}$, and the last equality is from the definition of the conditional expectation. It then follows the assumptions that $\{u^{*}_{j}:j\in M_{0}\}$ are independent, and $W_{i \cdot }^{\top }$ and *u*^∗^ are independent that

6 $$ {\begin{aligned} \mathbb{E}_{W_{i \cdot}}[\mathbb{E}_{u^{*}}(W_{i \cdot}u^{*})^{2}|W_{i \cdot}]&= \mathbb{E}_{W_{i \cdot}}\left\{\mathbb{E}_{u^{*}}\left[\left(\sum_{j\in M_{0}}W_{ij}u_{j}^{*}\right)^{2}{|}W_{i \cdot}\right]\right\}\\ &=\mathbb{E}_{W_{i \cdot}}\left\{\mathbb{E}_{u^{*}}\left[\sum_{j\in M_{0}}\left(W_{ij}u_{j}^{*}\right)^{2}{|}W_{i \cdot}\right]\right\}. \end{aligned}}  $$

By the assumption that for *j*∈*M*_0_, $\mathbb {E}\left (u^{*}_{j}\right)=0$ and $\text {Var}(u^{*}_{j})=\sigma _{u}^{2}$, one has that $\mathbb {E}_{u^{*}}\left [(u^{*}_{j})^{2}\right ]=\sigma _{u}^{2}$. Then substituting Eq. 6 into Eq. 5, we obtain that

7 $$ {\begin{aligned} \tau^{2}&\,=\,\mathbb{E}_{W_{i \cdot}}\left\{\mathbb{E}_{u^{*}}\left[\sum_{j\in M_{0}}\left(W_{ij}u_{j}^{*}\right)^{2}{|}W_{i \cdot}\right]\right\}\,=\,\mathbb{E}_{W_{i \cdot}}(\sigma_{u}^{2}\sum_{j\in M_{0}}W_{ij}^{2})\,=\,\sigma_{u}^{2}\sum_{j\in M_{0}}\mathbb{E}_{W_{i \cdot}}(W_{ij}^{2}). \end{aligned}}  $$

Since {*W*_*ij*_:*j*∈*M*_0_} are a set of centralized and normalized random variables with zero mean and unit variance by Eq. 2, we have that $\sum _{j\in M_{0}}\mathbb {E}\left (W_{ij}^{2}\right)=\sum _{j\in M_{0}}\text {Var}\left (W_{ij}\right)=s$, and finally arrive at that $\tau ^{2}=s\sigma _{u}^{2}$. It then follows immediately from Eq. 3 and Eq. 4 that, $h_{\text {fixed}}^{*}=h_{\text {rand.}}^{*}$. The proof is complete.

### Implementation

In this subsection, we introduce our two-stage strategy to estimate heritability, which consists of a sparse regularization step followed by a variance estimation step.

Before a detailed description of the strategy, let us assume that the sample size *m* is even for simplicity. Then the original data set (*y*,*W*) is randomly split into two disjoint data sets (*y*^(1)^,*W*^(1)^) and (*y*^(2)^,*W*^(2)^) with equal samples. Without loss of generality, the following sparse regularization step is performed on (*y*^(1)^,*W*^(1)^) to reduce the model, while the variance estimation step is applied on (*y*^(2)^,*W*^(2)^). In doing so, it is guaranteed that sparse regularization and variance estimation are performed on independent samples. We explain the reason for using independent samples at the end of this section.

#### Sparse regularization

Recall the model Eq. 1, which is a seriously ill-conditioned linear system with far fewer samples than variables (SNPs). Thus there exists no unique solution for the effect vector, and the problem of nonidentifiability appears. Fortunately, with the sparse assumptions mentioned above, the popular and practical regularization technique is applicable, which has been extensively studied for high dimensional linear models in the past decade; see, e.g., [22–24] and references therein.

Since in reality, one has no prior knowledge on the amount of each effect, the sparse regularization technique is required to be flexible to both small and large effects. In this paper, we adopt the elastic net [24] as our sparse regularization method. More precisely, we solve the following optimization problem:

8 $$ \min_{u\in\mathbb{R}^{n}}\frac{1}{2m}\left\|y^{(1)}-W^{(1)}u\right\|_{2}^{2}+\alpha\lambda \left\|u \|_{1}+\frac{1-\alpha}{2}\lambda \|u\right\|_{2}^{2},  $$

where *α*∈(0,1] represents the weight of Lasso [23] versus ridge [25] regularization, and *λ*>0 is the regularization parameter providing a tradeoff between accuracy and sparsity.

Here the parameter *α* is used to adapt to different sparsity levels. For high sparsity level, it is chosen to approach 1, while for lower sparsity level, it is chosen to be smaller. Though the real genetic architecture of a given trait is generally unknown, some prior knowledge may be used to roughly determine the value of *α*. A suitable choice of *λ* is critical as its value might strongly affect the set of variables selected. We here proceed to use the *k*-fold cross-validation to reduce the influence of false variables, and choose suitable values for *α* and *λ*.

**Fig. 1:**
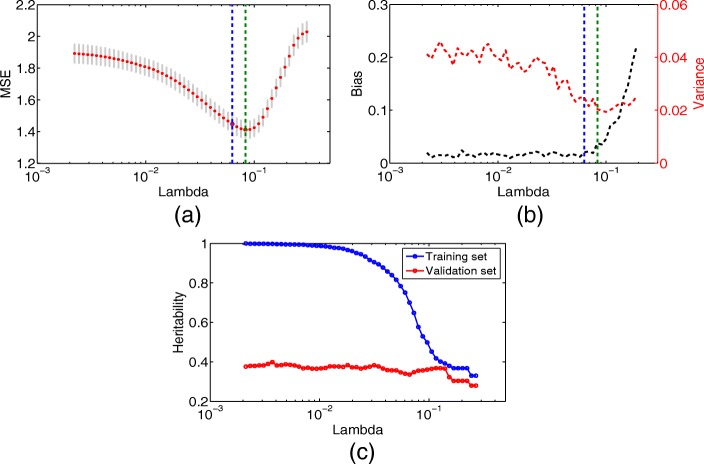
Illustrations of the proposed two-stage strategy. **a** 10-fold cross-validation to choose the most suitable regularization parameter; **b** The decomposition of bias and variance of the proposed strategy; **c** The explanation of the reason for using independent samples. Estimators in the validation set are obtained with independent samples, and estimators in the training set are obtained with non-independent samples

In practice, we fit the optimization problem Eq. 8 by implementing the MATLAB function “lasso” (https://www.mathworks.com/help/stats/lasso.html), which is designed for Lasso or elastic net regularization of linear models. Specifically, we first define a set *Ω* corresponding to the domain of *α*. Then for each *α*∈*Ω* fixed and a set of regularization parameters *λ* predefined, we perform 10-fold cross-validation and choose the smallest *λ* that is within one standard error of minimum prediction mean squared error (MSE), as is shown for instance in blue dashed line in Fig. 1a. Finally, we determine the value of *α* to be the one that meets the minimum prediction MSE across the set *Ω*. After the parameters *α* and *λ* have been determined, the selected model is denoted as $\hat {M}=\{j:\hat {u}_{j}\neq 0\}$, and the number of selected variables is $\hat {n}=|\hat {M}|$, where $\hat {u}$ is the optimal solution to Eq. 8.

#### Variance estimation

Now some certain variance estimation methods are applied to $\left (y^{(2)},W^{(2)}_{\hat {M}}\right)$. Recall that at this time, the sample size is only *m*/2.

For the fixed effect assumption, as $m/2>\hat {n}$ cannot be guaranteed, the problem might still be high dimensional. There are three notable works [26–28] considering variance estimation in high dimensional linear regression, among which the latter two rely strongly on the sparsity assumption on the model while the first one does not. Since the number of causal SNPs might vary from moderate (e.g., 10^2^∼10^3^) to large (e.g., 10^4^∼10^5^), the method must be stable with respect to the sparsity level. Moreover, as it is realistic that different SNPs are usually not independent, the method should also be capable of handling the case where there exist correlations between the SNPs. Therefore, we choose to use the method proposed in [26, section 4.2], which is based on the method-of-moment and is applicable to the correlated case. Two estimators for *τ*^2^ and $\sigma _{e}^{2}$ are constructed as follows:

$${\begin{aligned} \hat{\tau}^{2}&=-\frac{\hat{n}d_{1}^{2}}{m/2(m/2+1)d_{2}^{2}}\left\|y^{(2)}\right\|_{2}^{2}+\frac{d_{1}}{m/2(m/2+1)d_{2}}\left\|W^{(2)\top}_{\hat{M}} y^{(2)}\right\|_{2}^{2},\\ \hat{\sigma_{e}}^{2}&=\!\left(1\!+\frac{\hat{n}d_{1}^{2}}{(m/2+1)d_{2}}\right)\!\frac{1}{\hat{n}}\left\|y^{(2)}\right\|_{2}^{2}\!-\frac{d_{1}}{m/2(m/2+1)d_{2}}\left\|W^{(2)\top}_{\hat{M}} y^{(2)}\right\|_{2}^{2}, \end{aligned}} $$

where

$${}d_{1}=\frac{1}{\hat{n}}tr\left(\frac{1}{m/2}W^{(2)\top}_{\hat{M}} W^{(2)}_{\hat{M}}\right), $$

$${\begin{aligned} d_{2}=\frac{1}{\hat{n}}tr\left(\frac{1}{m/2}W^{(2)\top}_{\hat{M}} W^{(2)}_{\hat{M}}\right)^{2}-\frac{1}{\hat{n}m/2}\left(tr\left(\frac{1}{m/2}W^{(2)\top}_{\hat{M}} W^{(2)}_{\hat{M}}\right)\right)^{2}. \end{aligned}} $$

Note that when $m/2>\hat {n}$ and $W_{\hat {M}}$ has full rank, these two estimators are quite similar to the estimators obtained by ordinary least squares. Thus we arrive at a plug-in estimator for $h_{\text {fixed}}^{*}$:

$$\hat{h}_{\text{fixed}}=\frac{\hat{\tau}^{2}}{\hat{\tau}^{2}+\hat{\sigma_{e}}^{2}}. $$

For the random effect assumption, we simply apply the widely-used software GCTA [9], which implements the maximum likelihood method, with $\left (y^{(2)},W^{(2)}_{\hat {M}}\right)$ as the input to obtain estimators for variance components. Other tools such as BOLT-REML [11] or MEGHA [13] are of course applicable. The final estimator for $h_{\text {rand.}}^{*}$ is denoted as $\hat {h}_{\text {rand.}}$.

Since the true heritability always belongs to (0,1), once $\hat {h}_{\text {fixed}}$ or $\hat {h}_{\text {rand.}}$ is smaller than 0 or larger than 1, it is constrained to a value equal to 0.0001 or 0.9999, respectively. Nevertheless, as is shown by numerical results in the next section, performing a sparse regularization step first can perfectly restrict the obtained estimators to lie in (0,1).

To understand the behavior of the heritability estimator produced by our two-stage strategy, we make a decomposition of the bias and variance of the estimator. We only use $\hat {h}_{\text {rand.}}$ here so as to simplify the illustration, and $\hat {h}_{\text {fixed}}$ can also produce the same result. The corresponding result is displayed in Fig. 1b. Recall that *λ* is chosen to be the smallest one that is within one standard error of minimum MSE in section 2.2.1, as is shown in blue dashed line in Fig. 1a and b. We can see from Fig. 1b that when *λ* is too small and the selected model contains many redundant variables, though the heritability estimator is almost unbiased, its variance is large. Our choice of *λ* guarantees that the heritability estimator is not only almost unbiased but also with a smaller variance. The performance of our strategy will be demonstrated in detail in the next section.

Now let us turn to illustrate the reason for using independent samples in the proposed two-stage strategy. Assume that we are in the case where there are 10 causal SNPs out of total 10000 SNPs. Then Fig. 1c plots the heritability estimators versus the regularization parameter *λ* which represents the model selection process. We only use $\hat {h}_{\text {rand.}}$ here so as to simplify the illustration, and $\hat {h}_{\text {fixed}}$ can also produce the same result. The training set is used to select the model, and then variance estimation is completed on the training set and the validation set, respectively. Therefore, estimators in the training set are obtained with non-independent samples, and estimators in the validation set are obtained with independent samples. When the selected model contains too many redundant variables, its generalization ability is poor, and estimators produced by the training set are usually overestimated. As *λ* becomes larger, the selected model becomes more sparse, and the generalization ability of the selected model increases. Therefore, using samples independent of those used in model selection to estimate variance guarantees that even if the selected model is not sparse enough, the heritability won’t be overestimated. Otherwise, if model selection and variance estimation are done on the same sample set, the heritability is more likely to be overestimated. Hence, we suggest that model selection and variance estimation should be performed on independent samples to reduce overestimation.

### Simulated data

The simulated genotype data are generated via the R package “echoseq” (https://github.com/hruffieux/echoseq) [29]. Specifically, the genotype matrix *W* is generated with correlated columns based on generally accepted principles of population genetics (Hardy–Weinberg equilibrium, linkage disequilibrium, and natural selection). The sparse effect vector $u^{*}\in \mathbb {R}^{n}$ is generated by choosing *s* indices at random according to a $\mathcal {N}(0,1/s\mathbb {I}_{s})$ distribution, with different *s* being chosen for given *n*. The noise vector *e* is set as Gaussian with mean **0** and covariance matrix $\sigma _{e}^{2}\mathbb {I}_{m}$, with $\sigma _{e}^{2}$ representing the noise level. This generation process ensures that the simulated data behave like real genotype data. The observations *y* are then obtained via the model Eq. 1. The true value of heritability is approximated by

$$\tilde{h}^{*}=\frac{\left|Wu^{*}\right|_{2}^{2}/m}{\left|Wu^{*}\right|_{2}^{2}/m+\sigma_{e}^{2}}. $$

We see in the following simulations that the sample standard error of the approximation $\tilde {h}^{*}$ is so small that can be ignored.

### Real data from the IMAGEN project

Brain imaging scans were obtained from a cohort of 2089 adolescents (14.5±0.4 years old, 51% females) from the IMAGEN project (http://imagen-europe.com) using a standardised 3T, T1-weighted gradient echo protocol in eight European centres [30]. Genotype data were obtained using the Illumina 610-Quad and Illumina 660W-Quad chips, and then preprocessed using PLINK 1.90 (https://www.cog-genomics.org/plink2) [31]. We excluded SNPs that did not satisfy the following quality control criteria: genotype call rate ≥99*%*, minor allele frequency ≥1*%*, and Hardy-Weinberg equilibrium *P*≥1×10^−6^. After quality control, we finally used 225139 SNPs across the 22 autosomes genotyped on 1765 participants.

## Results

The purpose of this section is to carry out several experiments and demonstrate results on the heritability estimation problem for both simulated data and real data from the IMAGEN project. All experiments are performed in MATLAB R2014b and executed on a computer with the following configuration: Intel(R) Xeon(R) CPU E5-2630 v2, 12×2.60 GHz, 126 GB of RAM. The runtime for model selection is about 10 minutes and the required memory is about 8GB, with a data set including 1000 samples and 100000 SNPs, whose scale is close to that of real data. The following variance estimation step takes only a few seconds.

### Simulations on the fixed and random effect assumptions

To compare the influences of the fixed and random effect assumptions, the estimators $\hat {h}_{\text {fixed}}$ and $\hat {h}_{\text {rand.}}$ as well as the approximated true heritability $\tilde {h}^{*}$ are estimated under different noise levels $\sigma _{e}^{2}\in \{4,1,0.25\}$ and under the case where all the SNPs have nonzero effects for simplicity, that is *s*=*n* and *M*_0_={1,2,⋯,*n*}.

**Fig. 2:**
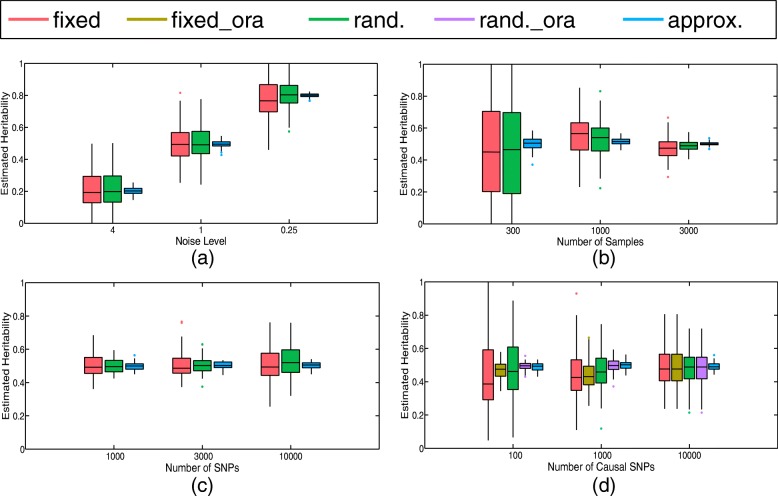
Boxplots of estimated heritability (100 replicates) under different simulation scenarios. Each plot presents results for one simulation scenario. **a** under different values of noise level (*m*=1000, *n*=10000); **b** under different values of the sample size (*s*=*n*=10000); **c** under different numbers of total SNPs (*m*=1000, *s*=*n*); **d** under different numbers of the causal SNPs (*m*=1000, *n*=10000). Here “fixed” refers to the estimator $\hat {h}_{\text {fixed}}$ with $\hat {M}=\{1,2,\cdots,n\}$, “fixed_ora” refers to the oracle estimator $\hat {h}_{\text {fixed}}$ with $\hat {M}=M_{0}$, “rand.” refers to the estimator $\hat {h}_{\text {rand.}}$ with $\hat {M}=\{1,2,\cdots,n\}$, and “rand._ora” refers to the oracle estimator $\hat {h}_{\text {rand.}}$ with $\hat {M}=M_{0}$. The approximation of the true heritability $\tilde {h}^{*}$ is denoted as “approx.”. The whiskers of each boxplot are the first and third quartiles

The corresponding boxplot is displayed in Fig. 2a. We can see from this figure that both the estimators $\hat {h}_{\text {fixed}}$ and $\hat {h}_{\text {rand.}}$ are almost unbiased, and that the approximation of the true heritability $\tilde {h}^{*}$ behaves well with small deviation that can be ignored. Moreover, it is also demonstrated that the fixed and random effect assumptions produce similar estimators.

### Simulations on the sample sizes and SNP sizes

To simplify our expositions, the following simulations are carried out under the case in which all the SNPs have nonzero effects, that is *s*=*n* and *M*_0_={1,2,⋯,*n*}. The noise level $\sigma _{e}^{2}$ is set equal to 1.

Firstly, we illustrate the performance of both estimators $\hat {h}_{\text {fixed}}$ and $\hat {h}_{\text {rand.}}$ under different sample sizes *m*∈{300,1000,3000}. The corresponding boxplot is displayed in Fig. 2b. We can see from this figure that the smaller the sample size, the larger the standard errors for both two estimators $\hat {h}_{\text {fixed}}$ and $\hat {h}_{\text {rand.}}$. In the case where the number of samples is relatively small, it is more likely to obtain many estimators reaching the boundaries 0 and 1, thus leading to estimations that are rather unreliable. Thus, when dealing with real GWAS data, the sample size should be as large as possible. This requirement can be easily satisfied for phenotypes like height and body mass index, while for phenotypes related to imaging genetics such as whole brain volume, it is not always the case. The lack of samples makes it a hard problem to estimate the heritability of these phenotypes.

Secondly, we illustrate the performance of both estimators $\hat {h}_{\text {fixed}}$ and $\hat {h}_{\text {rand.}}$ under different numbers of total SNPs *n*∈{1000,3000,10000}. The corresponding boxplot is displayed in Fig. 2c. We can see from this figure that the larger the number of SNPs, the larger the standard error of both estimators $\hat {h}_{\text {fixed}}$ and $\hat {h}_{\text {rand.}}$. This indicates that as the problem dimension gets larger, it becomes more difficult to obtain estimators with smaller standard errors. Thus in a typical GWAS, where the dimension is always thousands of hundreds while the number of samples cannot grow arbitrarily, the estimators should be treated carefully, since they may have large standard errors and lead to unreasonable results.

### Simulations on sparsity

To elucidate the importance of sparsity, both estimators $\hat {h}_{\text {fixed}}$ and $\hat {h}_{\text {rand.}}$ are estimated under different numbers of the causal SNPs *s*∈{100,1000,10000}. We use the oracle estimators corresponding to the fixed and random effect assumptions for comparisons, whose values are calculated via $\hat {h}_{\text {fixed}}$ and $\hat {h}_{\text {rand.}}$, respectively, with the oracle $\hat {M}=M_{0}$ known in advance. The noise level $\sigma _{e}^{2}$ is set equal to 1.

The corresponding boxplot is displayed in Fig. 2d. It has been shown in [21] that, when there are many nonzero entries contained in the effect vector, the estimators can still be unbiased even though the model is misspecified. However, the standard errors of these estimators are so large that cannot be accepted, as is shown in the case where *s*=100,1000. On the other hand, we can see from the oracle estimators that when the sparsity of *u*^∗^ is taken into consideration, the corresponding standard errors have been greatly reduced, resulting in more reliable estimations. In practice, since the set of causal SNPs is usually unknown, it is necessary to approximate the sparsity pattern of the effect vector *M*_0_ as close as possible before variance estimation.

### Simulations on the performance of the proposed strategy

**Fig. 3:**
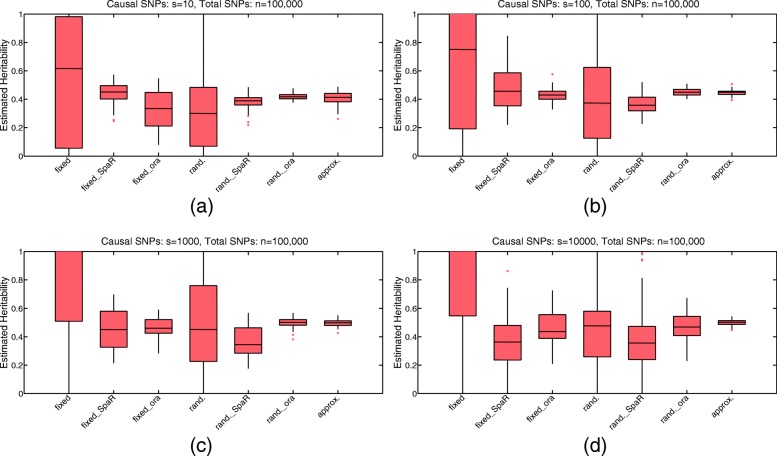
Boxplots of estimated heritability (100 replicates) under different problem sizes using the proposed strategy. **a** *s*=10, *m*=1000, *n*=100000; **b** *s*=100, *m*=1000, *n*=100000; **c** *s*=1000, *m*=1000, *n*=100000; **d** *s*=10000, *m*=1000, *n*=100000. Here “fixed” refers to the estimator $\hat {h}_{\text {fixed}}$ with $\hat {M}=\{1,2,\cdots,n\}$, “fixed_SpaR” refers to the estimator $\hat {h}_{\text {fixed}}$ with $\hat {M}$ given by our sparse regularization step, and “fixed_ora” refers to the oracle estimator $\hat {h}_{\text {fixed}}$ with $\hat {M}={M_{0}}$. “rand.” refers to the estimator $\hat {h}_{\text {rand.}}$ with $\hat {M}=\{1,2,\cdots,n\}$, “rand._SpaR” refers to the estimator $\hat {h}_{\text {rand.}}$ with $\hat {M}$ given by our sparse regularization step, and “rand._ora” refers to the oracle estimator $\hat {h}_{\text {rand.}}$ with $\hat {M}={M_{0}}$. The approximation of the true heritability $\tilde {h}^{*}$ is denoted as “approx.”. The whiskers of each boxplot are the first and third quartile

To illustrate the performance of the proposed two-stage strategy, both estimators $\hat {h}_{\text {fixed}}$ and $\hat {h}_{\text {rand.}}$ are estimated under different problem sizes. The oracle estimators are also used for comparisons with the oracle $\hat {M}=M_{0}$ known in advance. The noise level $\sigma _{e}^{2}$ is set equal to 1. The corresponding boxplots are displayed in Fig. 3.

We can see from Fig. 3 that, no matter in the highly sparse case or the more polygenic scenario, our two-stage strategy improves the performance of these estimators in the sense that the corresponding standard errors have been reduced considerably compared to those obtained without considering the sparsity structure. Moreover, when the sparsity level of underlying model is high, as displayed in Fig. 3a and b, our strategy is so impressive that it produces estimators performing as well as the oracle estimators, especially under the random effect assumption. In addition, we find that when there exist correlations between the SNPs and the problem dimension *n* is high (e.g., *n*=100000), the performance of the estimator $\hat {h}_{\text {fixed}}$ without considering the sparsity is somewhat undesirable in the sense that the standard error is too large to be acceptable, while the sparse regularization step reduces the standard error considerably. This result implies that our method is robust in the presence of correlations between the columns of *W*, and can be applied to the cases where LD exists.

### Simulations on real data from the IMAGEN project

We apply our two-stage strategy to estimate the heritability of height and the volume of neuroanatomical structures, specifically, the nucleus accumbens (Acc), amygdala (Amy), caudate nucleus (Ca), hippocampus (Hip), globus pallidus (Pa), putamen (Pu), and thalamus (Th).

As is widely-acknowledged that most human complex traits are generally polygenic and the corresponding heritability is largely captured by common SNPs [10, 32], the sparsity level cannot be too high in reality. Therefore, in the sparse regularization stage, we set the parameter *α*∈{3×10^−5^,10^−4^,3×10^−4^,10^−3^} in Eq. 8. In the variance estimation stage, the heritability is estimated under the random effect assumption. The standard error of the estimated heritability is approximated using the delta method [33]. The final results are displayed in Tab. 1 with the original results displayed in Additional file 1: Table S1. As far as we know, the heritability of these phenotypes from the IMAGEN project has also been estimated in [17] using GCTA, so Tab. 1 also includes their results for comparison.

**Table 1:** Heritability of height and the volume of neuroanatomical structures estimated from the IMAGEN project

Phenotype	SpaR		Toro GCTA	
	Heritability	Standard error	Heritability	Standard error
Height	0.48	0.18	0.53	0.23
Acc	0.30	0.11	0.52	0.23
Amy	0.43	0.21	0.45	0.23
Ca	0.06	0.15	0.16	0.23
Hip	0.70	0.19	0.53	0.23
Pa	0.21	0.14	0.31	0.23
Pu	0.50	0.19	0.54	0.23
Th	0.29	0.10	0.22	0.24

“SpaR” is used to denote results obtained by our two-stage strategy, and “Toro GCTA" is used to stand for results obtained in [17] by GCTA

We can see from Tab. 1 that the heritability estimated by our two-stage strategy is consistent with that reported in [17] on the same data set, with a considerably smaller standard error. This is especially the case for the volumes of Acc, Ca, Pa, and Th, where the corresponding standard error has been greatly reduced. In a word, our strategy can not only provide accurate estimations but also improve the reliability of the estimators in the sense that the standard error is reduced.

In addition to demonstrating the performance of our strategy, we analyse the heritability of average cortical thickness measures in 68 regions of interest (ROIs; 34 ROIs per hemisphere) defined by the Desikan-Killiany atlas [34]. The corresponding results are shown in Additional file 1: Table S2. Many estimators obtained using GCTA reach the boundaries (i.e., 0.0001 or 0.9999), which is of course unreasonable, while our strategy overcomes this obstacle to some extent in the sense that most of the estimators are perfectly restricted to the boundary set, leading to more stable and reliable results.

## Discussion

In this paper, we compared the fixed and random effect assumption in detail from both theoretical and practical aspects. In the theoretical aspect, we proved that the definitions of heritability are equivalent under mild conditions for both the fixed and random effect assumptions. In the practical aspect, our results demonstrated that both assumptions worked well, and produced similar estimators. However, when there exist correlations between the SNPs and the problem dimension *n* is high (e.g., *n*=100000), the performance of the estimator $\hat {h}_{\text {fixed}}$ is quite undesirable. Therefore, we recommended that $\hat {h}_{\text {rand.}}$ should be used in the real data analysis.

In modern GWASs, it has been pointed out in [18, 19, 35] that the sparsity structure usually exists in the ultrahigh dimensional genomic data. And our results on simulated data demonstrated that when the sparsity is considered, the standard errors of the heritability estimators had been greatly reduced (Fig. 2d). Therefore, it is quite necessary to take the sparsity structure into consideration and remove the redundant SNPs which are not related to the phenotype in heritability analyses. In practice, the set of causal SNPs is usually unknown, one needs to approximate the sparsity pattern as close as possible before variance estimation.

We proposed a two-stage strategy by first performing sparse regularization using cross-validated elastic net to select the model, and then applying certain variance estimation methods on the reduced model. Due to the fact that in the context of GWASs, there always exists a strong correlation between the explanatory variables (i.e., the SNPs) [36], attention is needed to the potential correlation structure between the SNPs when selecting the model. The elastic net [24] is especially powerful in the case where the pairwise correlations between variables may be high, and is more flexible to different sparsity levels. Moreover, the special structure of its regularization term, which is a linear combination of the Lasso [23] and the ridge [25] regression, enables one to simultaneously consider and balance two competing hypotheses that are usually used for explaining the underlying genetic architecture of human complex traits: common disease-rare variant hypothesis and common disease-common variant hypothesis [37], which address that, for some complex traits heritability may be explained by a small number of rare variants each with a large effect, while for other traits it may be explained by a large number of common variants with small effects. In a word, the elastic net can jointly balance the very sparse case and the more polygenic case.

Results from simulated data implied that our strategy produced estimators with considerably smaller standard errors than those obtained via methods without considering the sparsity (Fig. 3), leading to more reliable results for explanations. Moreover, we found that the performance of our strategy is more impressive when the sparsity level is high, in the sense that estimators obtained by our strategy behaves as well as the oracle estimators (Fig. 3a and b). This result points out a new prospect to analyse the complex genetic structure of some diseases that are caused by a few SNPs. Results from real data achieved estimations for the heritability of human height as well as the volumes of some neuroanatomical structures, which are consistent with former works [10, 17, 32] with smaller standard errors. In contemporary genomics, the sample size is usually limited due to physical or economical constraints, which is especially the case for brain imaging phenotypes. Therefore, our results show the promising future that reliable estimations can still be obtained with even a relatively restricted sample size.

While we are working on this paper, we became aware of an independent work [38]. Our contributions are substantially different from theirs, in that we perform variance estimation on a sample set independent of that used for model selection so as to avoid overestimation, while their variable selection and variance estimation steps are done on the same sample set. In addition, our sparse regularization technique is the elastic net, which is applicable in both the very sparse case and the more polygenic scenario, whereas they perform the variable selection through the sure independence screening approach followed by a Lasso criterion, resulting in a highly sparse model.

## Conclusion

We have considered the potential sparse structure of GWAS data, and proposed a two-stage strategy to produce reliable heritability estimations. Results on simulated data and real data demonstrate the promising future of our strategy for ultrahigh dimensional heritability analyses with even a relatively restricted sample size. Due to the fact that model selection consistency cannot be achieved unless certain strong conditions are satisfied (see, e.g., [39, 40]), the estimated heritability is actually the genetic variance attributed to the selected SNPs, and thus is indeed a lower bound for SNP heritability. Future directions of research may generalize our strategy to more precise models that can capture other underlying sophisticated structures of human complex traits, such as gene-gene and gene-environment interactions, to provide better estimations for heritability. In addition, it would be interesting to use our strategy in gene discovery and prediction analyses of complex traits.

## Additional file

Additional file 1 Additional estimation results using the proposed strategy. (PDF 242 kb)

## Abbreviations

Acc: Nucleus accumbens
Amy: Amygdala
Ca: Caudate nucleus
GCTA: Genome-wide complex trait analysis
GWAS: Genome-wide association study
Hip: Hippocampus
LD: Linkage disequilibrium
LMM: Linear mixed model
MEGHA: Massively expedited genome-wide heritability analysis
MSE: Mean squared error
Pa: Globus pallidus
Pu: Putamen
QTL: Quantitative trait locus
ROI: Region of interest
SNP: Single nucleotide polymorphism
Th: Thalamus

## References

[1] Falconer DS (1975). Introduction to Quantitative Genetics.

[2] Speed D, Cai N, Johnson MR, Nejentsev S, Balding DJ, Consortium U (2017). Reevaluation of SNP heritability in complex human traits. Nat Genet.

[3] Visscher PM, Medland SE, Ferreira MAR, Morley KI, Zhu G, Cornes BK, Montgomery GW, Martin NG (2006). Assumption-free estimation of heritability from genome-wide identity-by-descent sharing between full siblings. PLoS Genet.

[4] Vinkhuyzen AAE, Wray NR, Yang J, Goddard ME, Visscher PM (2013). Estimation and partition of heritability in human populations using whole-genome analysis methods. Annu Rev Genet.

[5] Gudbjartsson DF, Walters GB, Thorleifsson G, Stefansson H, Halldorsson BV, Zusmanovich P, Sulem P, Thorlacius S, Gylfason A, Steinberg S (2008). Many sequence variants affecting diversity of adult human height. Nat Genet.

[6] Weedon MN, Lango H, Lindgren CM, Wallace C, Evans DM, Mangino M, Freathy RM, Perry JR, Stevens S, Hall AS (2008). Genome-wide association analysis identifies 20 loci that influence adult height. Nat Genet.

[7] Maher B (2008). Personal genomes: The case of the missing heritability. Nat News.

[8] Manolio TA, Collins FS, Cox NJ, Goldstein DB, Hindorff LA, Hunter DJ, McCarthy MI, Ramos EM, Cardon LR, Chakravarti A (2009). Finding the missing heritability of complex diseases. Nature.

[9] Yang J, Lee SH, Goddard ME, Visscher PM (2011). GCTA: A tool for genome-wide complex trait analysis. Am J Hum Genet.

[10] Yang J, Benyamin B, McEvoy BP, Gordon S, Henders AK, Nyholt DR, Madden PA, Heath AC, Martin NG, Montgomery GW (2010). Common SNPs explain a large proportion of the heritability for human height. Nat Genet.

[11] Loh P-R, Bhatia G, Gusev A, Finucane HK, Bulik-Sullivan BK, Pollack SJ, de Candia TR, Lee SH, Wray NR, Kendler KS (2015). Contrasting genetic architectures of schizophrenia and other complex diseases using fast variance-components analysis. Nat Genet.

[12] Moser G, Lee SH, Hayes BJ, Goddard ME, Wray NR, Visscher PM (2015). Simultaneous discovery, estimation and prediction analysis of complex traits using a Bayesian mixture model. PLoS Genet.

[13] Ge T, Nichols TE, Lee PH, Holmes AJ, Roffman JL, Buckner RL, Sabuncu MR, Smoller JW (2015). Massively expedited genome-wide heritability analysis (MEGHA). Proc Natl Acad Sci.

[14] Lynch M, Walsh B (1998). Genetics and Analysis of Quantitative Traits.

[15] Bryant C, Giovanello KS, Ibrahim JG, Chang J, Shen D, Peterson BS, Zhu HT (2013). Mapping the genetic variation of regional brain volumes as explained by all common SNPs from the ADNI study. PLoS One.

[16] Kumar SK, Feldman MW, Rehkopf DH, Tuljapurkar S (2016). Limitations of GCTA as a solution to the missing heritability problem. Proc Natl Acad Sci.

[17] Toro R, Poline J-B, Huguet G, Loth E, Frouin V, Banaschewski T, Barker GJ, Bokde A, Büchel C, Carvalho FM (2015). Genomic architecture of human neuroanatomical diversity. Mol Psychiatry.

[18] Ripke S, Neale BM, Corvin A, Walters JTR, Farh K-H, Holmans PA, Lee P, Bulik-Sullivan B, Collier DA, Huang H (2014). Biological insights from 108 schizophrenia-associated genetic loci. Nature.

[19] Stahl EA, Wegmann D, Trynka G, Gutierrez-Achury J, Do R, Voight BF, Kraft P, Chen R, Kallberg HJ, Kurreeman FAS (2012). Bayesian inference analyses of the polygenic architecture of rheumatoid arthritis. Nat Genet.

[20] Patterson HD, Thompson R (1971). Recovery of inter-block information when block sizes are unequal. Biometrika.

[21] Jiang JM, Li C, Paul D, Yang C, Zhao HY (2016). On high-dimensional misspecified mixed model analysis in genome-wide association study. Ann Stat.

[22] Hu YH, Li C, Meng KW, Qin J, Yang XQ (2017). Group sparse optimization via *ℓ*_*p*,*q*_ regularization. J Mach Learn Res.

[23] Tibshirani R (1996). Regression shrinkage and selection via the Lasso. J Royal Stat Soc B.

[24] Zou H, Hastie T (2005). Regularization and variable selection via the elastic net. J Royal Stat Soc B.

[25] Hoerl AE, Kennard RW (1970). Ridge regression: Biased estimation for nonorthogonal problems. Technometrics.

[26] Dicker LH (2014). Variance estimation in high-dimensional linear models. Biometrika.

[27] Fan JQ, Guo SJ, Hao N (2012). Variance estimation using refitted cross-validation in ultrahigh dimensional regression. J Royal Stat Soc B.

[28] Sun TN, Zhang C-H (2012). Scaled sparse linear regression. Biometrika.

[29] Ruffieux H, Davison AC, Hager J, Irincheeva I (2017). Efficient inference for genetic association studies with multiple outcomes. Biostatistics.

[30] Schumann G, Loth E, Banaschewski T, Barbot A, Barker G, Büchel C, Conrod PJ, Dalley JW, Flor H, Gallinat J (2010). The IMAGEN study: reinforcement-related behaviour in normal brain function and psychopathology. Mol Psychiatry.

[31] Chang CC, Chow CC, Tellier LCAM, Vattikuti S, Purcell SM, Lee JJ (2015). Second-generation PLINK: Rising to the challenge of larger and richer datasets. Gigascience.

[32] Wood AR, Esko T, Yang J, Vedantam S, Pers TH, Gustafsson S, Chu AY, Estrada K, Luan J, Kutalik Z (2014). Defining the role of common variation in the genomic and biological architecture of adult human height. Nat Genet.

[33] Hohls T (1996). Setting confidence limits to genetic parameters estimated by restricted maximum likelihood analysis of North Carolina design II experiments. Heredity.

[34] Desikan RS, Ségonne F, Fischl B, Quinn BT, Dickerson BC, Blacker D, Buckner RL, Dale AM, Maguire RP, Hyman BT (2006). An automated labeling system for subdividing the human cerebral cortex on MRI scans into gyral based regions of interest. Neuroimage.

[35] Yazdani A, Boerwinkle E (2015). Rare variants analysis using penalization methods for whole genome sequence data. BMC Bioinform.

[36] Botta V, Louppe G, Geurts P, Wehenkel L (2014). Exploiting SNP correlations within random forest for genome-wide association studies. PLoS One.

[37] Gibson G (2012). Rare and common variants: Twenty arguments. Nat Rev Genet.

[38] Bonnet A, Lévy-Leduc C, Gassiat E, Toro R, Bourgeron T (2018). Improving heritability estimation by a variable selection approach in sparse high dimensional linear mixed models. J Royal Stat Soc C.

[39] Meinshausen N, Yu B (2009). Lasso-type recovery of sparse representations for high-dimensional data. Ann Stat.

[40] Zhao P, Yu B (2006). On model selection consistency of Lasso. J Mach Learn Res.

