# Supplementary material for: Reliable heritability estimation using sparse regularization in ultrahigh dimensional genome-wide association studies
Source: BMC Bioinformatics. 2019 Apr 30;20:219. doi: 10.1186/s12859-019-2792-7 (PMC6492418; doi:10.1186/s12859-019-2792-7)
Supplement: Supplementary file 1 — Additional estimation results using the proposed strategy. (PDF 242 kb) [file 12859_2019_2792_MOESM1_ESM.pdf]

Table S1. Original estimation results of height and the volume of neuroanatomical structures using the proposed strategy.

Phenotype	SpaR		GCTA	
	heritability	standard error	heritability	standard error
Height	0.4778	0.1844	0.6733	0.2112
Acc	0.2953	0.1057	0.5335	0.2327
Amy	0.4282	0.2078	0.4819	0.2113
Ca	0.0606	0.1454	0.1861	0.2360
Hip	0.6976	0.1859	0.9999	0.2133
Pa	0.2081	0.1402	0.1728	0.2201
Pu	0.4988	0.1879	0.5061	0.2441
Th	0.2918	0.1023	0.4078	0.2205

$\alpha = 3 \times 10^{-5}$				
Phenotype	heritability	standard error	number of SNPs selected	MSE
Height	0.6216	0.2190	161,342	0.9464
Acc	0.6767	0.1922	158,919	1.1590
Amy	0.4282	0.2078	152,011	0.8718
Ca	0.3531	0.2108	156,070	0.9692
Hip	0.8618	0.2152	153,669	0.9663
Pa	0.3159	0.2015	153,941	0.9230
Pu	0.6512	0.2198	157,911	1.0417
Th	0.5923	0.2174	155,743	0.8949
$\alpha = 1 \times 10^{-4}$				
Phenotype	heritability	standard error	number of SNPs selected	MSE
Height	0.4778	0.1844	101,974	0.9355
Acc	0.6664	0.1766	103,084	1.1597
Amy	0.3586	0.1811	99,336	0.8720
Ca	0.2505	0.1811	99,941	0.9722
Hip	0.6976	0.1859	94,427	0.9635
Pa	0.2703	0.1779	97,640	0.9238
Pu	0.4988	0.1879	101,975	1.0354
Th	0.5102	0.1872	102,371	0.8906
$\alpha = 3 \times 10^{-4}$				
Phenotype	heritability	standard error	number of SNPs selected	MSE
Height	0.3252	0.1471	57,556	0.9456
Acc	0.4981	0.1478	61,167	1.1663
Amy	0.0352	0.1169	39,181	0.8740
Ca	0.0606	0.1454	57,536	0.9656
Hip	0.5630	0.1470	52,071	0.9643
Pa	0.2081	0.1402	49,802	0.9162

Pu	0.3450	0.1503	57,520	1.0356
Th	0.3609	0.1309	41,130	0.8891
$\alpha = 1 \times 10^{-3}$				
Phenotype	heritability	standard error	number of SNPs selected	MSE
Height	0.0630	0.1036	24,842	0.9390
Acc	0.2953	0.1057	26,331	1.1589
Amy	0.0001	0.0915	21,172	0.8721
Ca	0.0001	0.1044	24,800	0.9688
Hip	0.2185	0.0942	22,556	0.9716
Pa	0.0723	0.1001	23,862	0.9163
Pu	0.3288	0.1114	25,490	1.0380
Th	0.2918	0.1023	21,879	0.8827

Table S2. Heritability analyses of average cortical thickness measures in 68 ROIs using the proposed strategy.

ROI	SpaR		GCTA	
	heritability	standard error	heritability	standard error
Left MeanThickness	0.4079	0.1486	0.5256	0.1723
Left bankssts	0.3753	0.1534	0.3795	0.1888
Left caudalanteriorcingulate	0.2379	0.1328	0.3895	0.1692
Left caudalmiddlefrontal	0.1889	0.1344	0.3469	0.1780
Left cuneus	0.3454	0.1385	0.3321	0.1704
Left entorhinal	0.0752	0.1285	0.2807	0.1799
Left frontalpole	0.7826	0.1487	0.9999	0.1915
Left fusiform	0.1996	0.1206	0.6158	0.1923
Left inferiorparietal	0.3002	0.1419	0.7001	0.1956
Left inferiortemporal	0.2211	0.1434	0.3626	0.1719
Left insula	0.1814	0.1158	0.4219	0.1777
Left isthmuscingulate	0.3108	0.1359	0.5302	0.1800
Left lateraloccipital	0.6175	0.1433	0.9999	0.2015
Left lateralorbitofrontal	0.9938	0.1430	0.9999	0.1673
Left lingual	0.0180	0.1389	0.0001	0.1572
Left medialorbitofrontal	0.9045	0.1358	0.9999	0.1816
Left middletemporal	0.2524	0.1444	0.3417	0.1657
Left paracentral	0.3327	0.1369	0.4796	0.1658
Left parahippocampal	0.1594	0.1344	0.2626	0.1790
Left parsopercularis	0.4076	0.1403	0.6176	0.1838
Left parsorbitalis	0.7992	0.1549	0.9999	0.1989
Left parstriangularis	0.7354	0.1471	0.9999	0.1892
Left pericalcarine	0.3113	0.1233	0.5148	0.1813
Left postcentral	0.2237	0.1365	0.5567	0.1818
Left posteriorcingulate	0.6640	0.1562	0.9999	0.2047
Left precentral	0.1763	0.1309	0.2463	0.1643

Left precuneus	0.1426	0.1305	0.4081	0.1732
Left rostralanteriorcingulate	0.1327	0.1211	0.2308	0.1582
Left rostralmiddlefrontal	0.9765	0.1602	0.9999	0.1863
Left superiorfrontal	0.7835	0.1436	0.9999	0.1886
Left superiorparietal	0.5012	0.1490	0.6936	0.1898
Left superiortemporal	0.2578	0.1337	0.3707	0.1649
Left supramarginal	0.3769	0.1330	0.7825	0.2068
Left temporalpole	0.0093	0.1319	0.2261	0.1676
Left transversetemporal	0.5476	0.1446	0.9999	0.2170
Right MeanThickness	0.3612	0.1278	0.5256	0.1723
Right bankssts	0.1203	0.1429	0.3795	0.1888
Right caudalanteriorcingulate	0.2851	0.1365	0.3895	0.1692
Right caudalmiddlefrontal	0.2691	0.1540	0.3469	0.1780
Right cuneus	0.0001	0.1295	0.3321	0.1704
Right entorhinal	0.2156	0.1520	0.2807	0.1799
Right frontalpole	0.7988	0.1450	0.9999	0.1915
Right fusiform	0.5053	0.1567	0.6158	0.1923
Right inferiorparietal	0.3945	0.1320	0.7001	0.1956
Right inferiortemporal	0.2617	0.1297	0.3626	0.1719
Right insula	0.3487	0.1492	0.4219	0.1777
Right isthmuscingulate	0.0898	0.1175	0.5302	0.1800
Right lateraloccipital	0.6832	0.1560	0.9999	0.2015
Right lateralorbitofrontal	0.9999	0.1562	0.9999	0.1673
Right lingual	0.0001	0.1387	0.0001	0.1572
Right medialorbitofrontal	0.9934	0.1476	0.9999	0.1816
Right middletemporal	0.2881	0.1453	0.3417	0.1657
Right paracentral	0.2955	0.1298	0.4796	0.1658
Right parahippocampal	0.1908	0.1375	0.2626	0.1790
Right parsopercularis	0.4018	0.1376	0.6176	0.1838
Right parsorbitalis	0.7456	0.1529	0.9999	0.1989
Right parstriangularis	0.8466	0.1578	0.9999	0.1892
Right pericalcarine	0.1705	0.1370	0.5148	0.1813
Right postcentral	0.2705	0.1508	0.5567	0.1818
Right posteriorcingulate	0.6668	0.1488	0.9999	0.2047
Right precentral	0.4682	0.1606	0.2463	0.1643
Right precuneus	0.2714	0.1497	0.4081	0.1732
Right rostralanteriorcingulate	0.1187	0.1399	0.2308	0.1582
Right rostralmiddlefrontal	0.9201	0.1516	0.9999	0.1863
Right superiorfrontal	0.8798	0.1581	0.9999	0.1886
Right superiorparietal	0.3988	0.1490	0.6936	0.1898
Right superiortemporal	0.2152	0.1313	0.3707	0.1649
Right supramarginal	0.4427	0.1533	0.7825	0.2068
Right temporalpole	0.3179	0.1450	0.2261	0.1676

Right transversetemporal	0.6495	0.1522	0.9999	0.2170
